# Estimation of axial curvature of anterior sclera: correlation between axial length and anterior scleral curvature as affected by angle kappa

**DOI:** 10.1186/s12886-016-0355-5

**Published:** 2016-10-07

**Authors:** Sang-Mok Lee, Hyuk Jin Choi, Heejin Choi, Mee Kum Kim, Won Ryang Wee

**Affiliations:** 1 Department of Ophthalmology, Hallym University Sacred Heart Hospital, Hallym University College of Medicine, Anyang, Republic of Korea; 2 Laboratory of Corneal Regenerative Medicine and Ocular Immunology, Seoul Artificial Eye Center, Seoul National University Hospital Biomedical Research Institute, Seoul, Republic of Korea; 3 Department of Ophthalmology, Seoul National University College of Medicine, 101 Daehang-ro, Jongno-gu, Seoul 110-744 Republic of Korea; 4 Institute for Medical Engineering and Science (IMES), Massachusetts Institute of Technology, Cambridge, MA USA

**Keywords:** Sclera, Biometry, Radius, Optical coherence tomography, Regression analysis, Contact lenses

## Abstract

**﻿Background:**

Though the development and fitting of scleral contact lenses are expanding steadily, there is ﻿no simple method to provide scleral metrics for scleral contact lens fitting yet. The aim of this study was to establish formulae for estimation of the axial radius of curvature (ARC) of the anterior sclera using ocular biometric parameters that can be easily obtained with conventional devices.

**Methods:**

A semi-automated stitching method and a computational analysis tool for calculating ARC were developed by using the ImageJ and MATLAB software. The ARC of all the ocular surface points were analyzed from the composite horizontal cross-sectional images of the right eyes of 24 volunteers; these measurements were obtained using anterior segment optical coherence tomography for a previous study (AS-OCT; Visante). Ocular biometric parameters were obtained from the same volunteers with slit-scanning topography and partial coherence interferometry. Correlation analysis was performed between the ARC at 8 mm to the axis line (ARC[8]) and other ocular parameters (including age). With ARC obtained on several nasal and temporal points (7.0, 7.5, 8.0, 8.5, and 9.0 mm from the axis line), univariate and multivariate linear regression analyses were performed to develop a model for estimating ARC with the help of ocular biometric parameters.

**Results:**

Axial length, spherical equivalent, and angle kappa showed correlations with temporal ARC[8] (tARC[8]; Pearson’s *r* = 0.653, −0.579, and −0.341; *P* = 0.001, 0.015, and 0.015, respectively). White-to-white corneal diameter (WTW) and anterior chamber depth (ACD) showed correlation with nasal ARC[8] (nARC[8]; Pearson’s *r* = −0.492 and −0.461; *P* = 0.015 and 0.023, respectively). The formulae for estimating scleral curvatures (tARC, nARC, and average ARC) were developed as a function of axial length, ACD, WTW, and distance from the axis line, with good determinant power (72 − 80 %; SPSS ver. 22.0). Angle kappa showed strong correlation with axial length (Pearson’s *r* = −0.813, *P* <0.001), and the different correlation patterns of nasal and temporal ARC with axial length can be explained by the ocular surface deviation represented by angle kappa.

**Conclusions:**

Axial length, ACD, and WTW are useful parameters for estimating the ARC of the anterior sclera, which is important for the haptic design of scleral contact lenses. Angle kappa affects the discrepancies between the nasal and temporal scleral curvature.

**Electronic supplementary material:**

The online version of this article (doi:10.1186/s12886-016-0355-5) contains supplementary material, which is available to authorized users.

## Background

The sclera is a dense, fibrous, and viscoelastic connective tissue that covers approximately 85 % of the surface area of the eyeball [1, 2]. The connective tissue matrix is composed of bundles of parallel-aligned collagen fibrils and provides the strength and resilience needed for optical stability during eye movement [1]. Although the sclera still remains an under-researched area, it has recently drawn attention in investigations of ocular disease and pathology because of the importance of scleral properties and biomechanics [1–3]. In addition, recent studies have attempted to measure scleral metrics by anterior segment optical coherence tomography (AS-OCT) [4–6].

The development and fitting of scleral contact lenses are expanding steadily owing to their unique therapeutic role in ocular surface diseases and corneal ectasia, with recent advancements in rigid contact lens materials [7, 8]. Unlike the fitting of smaller, conventional rigid gas permeable lenses that can be assisted by corneal metrics easily acquired using conventional imaging devices, the fitting of scleral lenses is still time-consuming and experience dependent because no conventional devices have yet been developed that can primarily provide scleral metrics [3, 9]. Therefore, the trial fitting of its haptic (scleral) part begins usually based on the corneal metrics, which is already well known to be poorly correlated with scleral metrics [3, 6]. To improve the difficult fitting process that limits wider use of scleral lenses, measurement of scleral metrics and post-fitting assessments using AS-OCT have drawn attention recently [3, 8, 10]. As one of these attempts, we have developed a manual method to calculate the axial radius of curvature (ARC), by 1) incorporating three horizontal cross-sectional AS-OCT images into a composite image using Microsoft PowerPoint (version 2007, Redmond, WA, USA), 2) identifying the *x* and *y* coordinates of six scleral points (three nasal and three temporal) on the composite image, and 3) calculating the ARC with a spreadsheet or radius of a best-fit circle with commercial image analysis software [9]. Although this method demonstrated excellent reliability and accuracy, it requires extensive training to get repetitive results. Further, it is time-consuming, and requires an expensive image processing software and an AS-OCT machine.

Therefore, the aim of this study was to establish a simpler and more automated method for calculating ARC of the anterior sclera using AS-OCT images and to develop formulae to estimate ARC using more easily measurable ocular biometric parameters.

## Methods

### Subjects

The images of the right eyes of healthy volunteers who were enrolled in our previous study were reanalyzed for the ARC using a newly developed automated method. The volunteers were enrolled after thorough history taking and a full ophthalmic examination [9]. The subjects with the following history and findings were excluded from enrollment: previous scleral or conjunctival diseases, ocular surgeries involving the sclera or conjunctiva, current conjunctival degenerative changes including pinguecula or pterygium, or current contact lens wearer.

Twenty-four Asian participants were enrolled, including 12 women and 12 men, with a mean age of 31.3 ± 6.5 years (Table 1). Only data from the right eye were used for the analysis. Subgroup analysis was performed by grouping the 24 eyes into four groups according to axial length, two groups according to white-to-white corneal diameter (WTW), and four groups according to anterior chamber depth (ACD), to demonstrate the changes in ARC in the wider nasal and temporal scleral areas (7.0, 7.5, 8.0, 8.5, and 9.0 mm from the axis line) according to the parameters.

**Table 1:** Mean values and ranges of the age and ocular biometric parameters

Parameter	Unit	Mean	SD	Range
Age	years	31.3	6.5	24 − 54
Sim Kmax	D	44.1	1.2	42.4 − 47.0
Sim Kmin	D	42.6	1.4	40.7 − 46.7
SE	D	−3.34	3.13	−8.88 − +1.13
WTW	mm	11.6	0.3	11.1 − 12.3
Thinnest corneal thickness	μm	530	42	477 − 607
ACD	mm	2.99	0.30	2.28 − 3.47
Angle Kappa	degree	4.3	1.3	1.68 − 6.39
Axial length	mm	25.0	1.5	21.9 − 27.6

*Sim Kmax* maximum simulated keratometric value, *Sim Kmin* minimum simulated keratometric value, *SE* spherical equivalent, *WTW* white-to-white corneal diameter, *ACD* anterior chamber depth from endothelium, *D* diopters, *SD* standard deviation

### Acquisition of the ocular biometric data and anterior segment tomographic images

After the full ophthalmic examination using a slit lamp, each subject underwent the following ocular biometric data measurements: refractive status (spherical equivalent) with an autorefractor/keratometer (RK-F1; Canon, Tokyo, Japan); angle kappa, simulated keratometric value (Sim K), WTW, thinnest corneal thickness, ACD (from endothelium) with slit-scanning topography (Orbscan II, Bausch and Lomb, Rochester, NY, USA); and axial length with partial coherence interferometry (IOLMaster Version 5.4; Carl Zeiss Meditech Inc., Dublin, CA, USA).

The cross-sectional ocular images obtained in the previous study from the same volunteers were used for a new analytical method [9]. Briefly, three horizontal images, each centered on the visual axis, temporal cornea, and nasal cornea were obtained by using AS-OCT (Visante OCT, Carl Zeiss Meditec Inc, Dublin, CA) with nasal and temporal external targets that were located approximately 15° from the primary position to align the three images on the same horizontal plane [9].

### Analysis of the scleral curvature

**Fig. 1:**
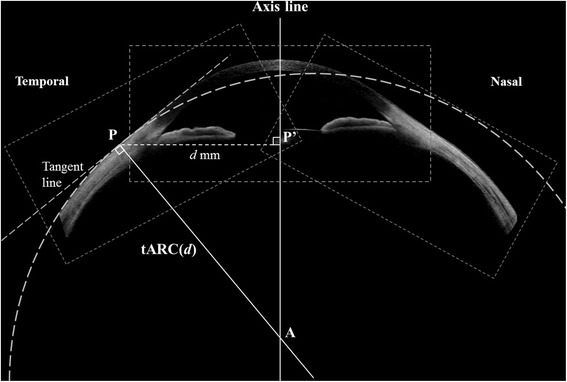
Concept of the axial radius of curvature (ARC) of the anterior sclera. The ARC of a certain ocular surface point is calculated on the basis of the following concept: for an ocular surface point (P) where the distance from the axis line is *d* (distance of PP’), a line can be drawn that passes point P and is perpendicular to the line tangential to the ocular surface at point P. Point A is the point where the perpendicular line meets the axis line. The distance of PA is ARC(*d*). If point P is temporal to the axis line, it will be designated as tARC(*d*). If the point is nasal to the axis line, it will be designated as nARC(*d*). The axis line is defined as a bright vertical flare line that passes the corneal apex and is parallel to the visual axis

To analyze the ARC in the sclera, composite center, nasal, and temporal tomographic images were needed because the current AS-OCT scanner provides images with a maximum width of 16 mm, which cannot include the cornea and enough scleral area together in a single image, which is mandatory to analyze axial curvatures by definition (Fig. 1).

After rotating the nasal and temporal horizontal images by using the image rotate function of a public domain Java-based image processing program ImageJ (Version 1.49p; National Institutes of Health, Bethesda, MD), the composite images were created semi-automatically using the MosaicJ plugin (Additional file 1: Figure S1). MosaicJ is a plugin for ImageJ that stitches overlapping images together with fine adjustments to correct for the small degrees of horizontal, vertical, and rotational misalignment [11].

**Fig. 2:**
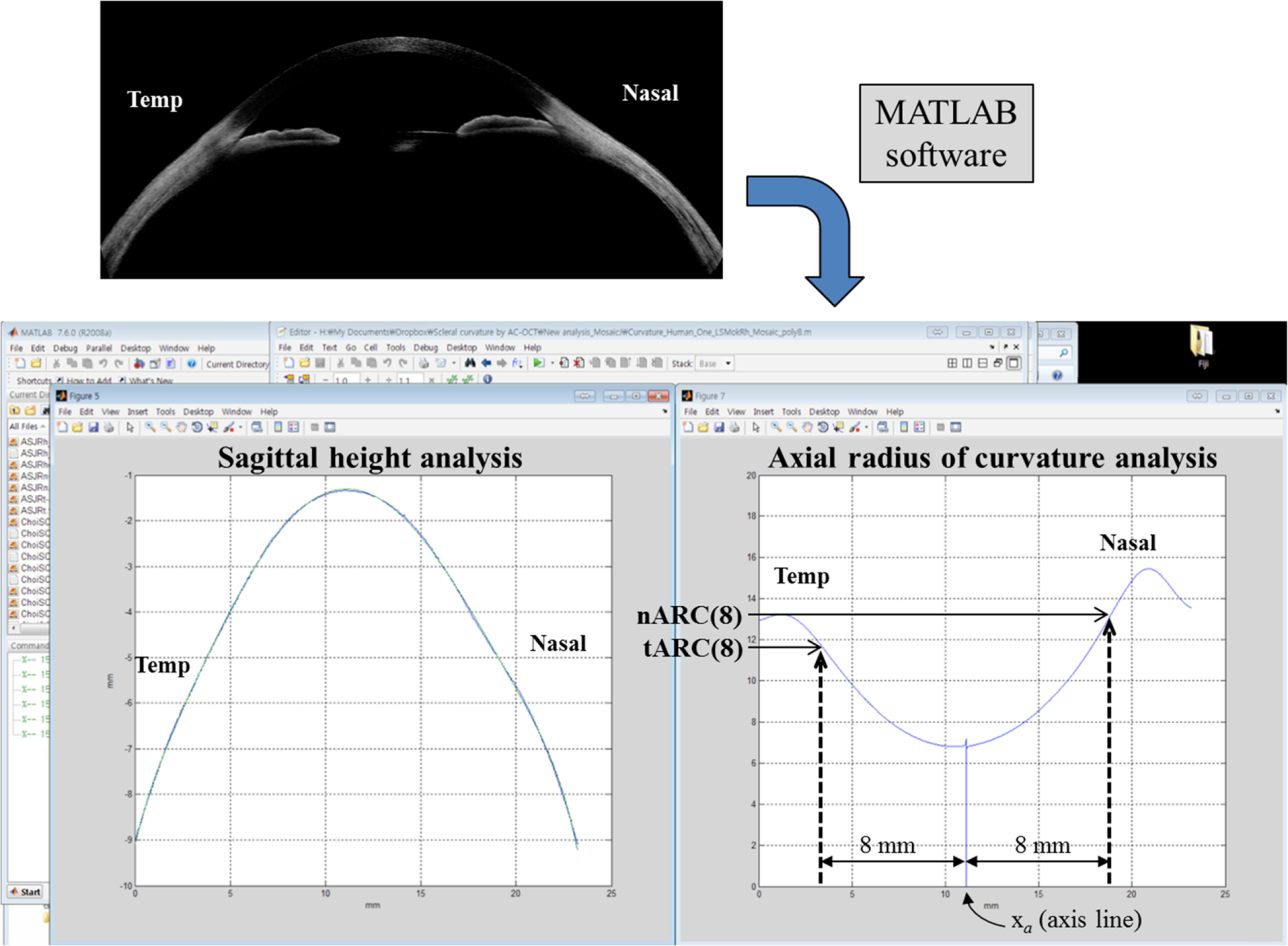
Analysis of the axial radius of curvature (ARC) from a composite anterior segment tomographic image. After removing the background noise, the shape of the outermost layer of the ocular surface was automatically extracted and curve-fitted using the image processing and optimization toolbox of MATLAB (MathWorks, Natick, MA) (*bottom left*). The axial curvature was then calculated mathematically as a function of the distance from the axis line (*d*, *bottom right*). The nARC[8] and tARC[8] were used for the correlation analysis

The concept of ARC is shown in Fig. 1, and the axis line was defined as a bright vertical flare line that passes the corneal apex and is parallel to the visual axis [9]. After manually removing the strong reflection background from the corneal apex of the composite image of the AS-OCT by using ImageJ, the ARC was automatically calculated with the image processing and optimization toolbox of MATLAB (MathWorks, Natick, MA). In other words, the image was preprocessed to remove background noise with the simple thresholding filter in MATLAB differentiating the intensity between the background noise and the signal from the ocular surface. The shape of the outermost layer of the ocular surface was subsequently extracted and curve-fitted using an eighth-order polynomial. The order of polynomial function was determined to identify the best approximation to the shape of the ocular surface. The ocular surface typically has three convex curves that correspond to temporal sclera, cornea, and nasal sclera. In addition, the shape of the ocular surface can be approximated as a smooth curve without any sharp point. Thus, the ocular surface has at least four inflection points, and the minimum polynomial order that satisfies this condition is six. We also noticed that the *R* ^2^ value further decreased as the order increased to higher even order and we chose the eighth order for this study. A mathematical formula based on the definition of the axial curvature was derived, and can be expressed as follows:

$$ {R}_A\left({x}_1\right)=d\sqrt{1+\frac{1}{f^{\hbox{'}}{\left({x}_1\right)}^2}},d=\left|{x}_1-{x}_a\right| $$

where *R*_A_ is the ARC, *x*_*a*_ is the position of the axis line, *f(x)* is the eighth-order polynomial representing the shape of the outermost layer of the cornea and sclera, and *f’(x)* represents the slope of the ocular surface at position *x* (Fig. 2). With this method, the ARC can be calculated for every pixel point of the ocular surface as a function of normal distance from the axis line (*d*, Fig. 1). Several points on the temporal and nasal sclera (*d* = 7.0, 7.5, 8.0, 8.5, and 9.0 mm) were selected for statistical analysis and model fitting. The ARC of a point where *d* equals 8 mm was used for the correlation analysis (nasal: nARC[8], temporal: tARC[8], and average: aARC[8], Fig. 2) because this point is approximately 1.8 − 2.4 mm from the limbus, which is clinically important for fitting scleral lenses and suction rings in LASIK as the center of the contact area [9, 12, 13].

### Statistical analyses

Paired t-tests were used for the comparison of nasal and temporal scleral curvatures. Mann-Whitney *U* tests and one-way analysis of variance (ANOVA) were used for the subgroup analysis. The Pearson correlation analysis was used to reveal the correlation among the ocular biometric parameters, including ARC and age. Although age is a demographic parameter, it was included in the correlation and regression analyses owing to its known correlation with the tangential scleral curvature [5]. Univariate and multivariate linear regression analyses were performed to develop the model for estimating ARC (forward stepwise method: probability-of-F-to-enter ≤0.05 and probability-of-F-to-remove ≥0.10, excluding the outliers over 2 standard deviations). The following input variables were used in the univariate linear regression analysis: age, spherical equivalent, maximum and minimum Sim K, WTW, thinnest corneal thickness, ACD, angle kappa, axial length, and *d*. The best-fit model for tARC, nARC, and aARC were determined using multivariate linear regression analysis with the input variables identified as significant in the univariate linear regression analysis (criteria: *P* <0.05). All the analyses were performed using SPSS Version 22.0 for Windows (SPSS Inc., Chicago, IL), and a *P* value <0.05 was considered statistically significant.

## Results

### Comparison of nasal and temporal scleral curvatures

**Fig. 3:**
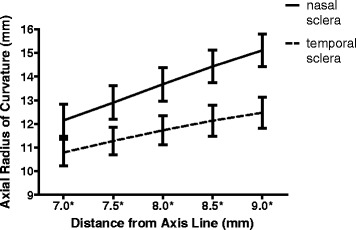
Comparison of the nasal and temporal scleral curvature. The mean nasal axial radius of curvature (nARC) values were significantly higher than the corresponding temporal ARC (tARC) values at all the 5 points analyzed (*d* = 7.0, 7.5, 8.0, 8.5, and 9.0 mm). The differences in nARC and tARC gradually increased as the distance from the axis line (*d*) increased. * *P* <0.001, Paired samples *t*-test

The curvature of the nasal sclera was significantly flatter than that of the temporal sclera, where *d* equals 7.0, 7.5, 8.0, 8.5, and 9.0 mm (*P* <0.001 for every point, Paired samples *t*-test; Fig. 3). As a center of this area, the nARC[8] (13.68 ± 0.71 mm) was significantly flatter than the tARC[8] (11.73 ± 0.61 mm) which was similar to our previous report but with reduced variation (nasal: 13.33 ± 1.12 mm, temporal: 12.32 ± 0.77 mm) [9]. In addition, the differences in nasal and temporal scleral curvatures gradually increased as the distance from the axis line increased.

### Correlation between scleral curvature and ocular biometric parameters including age

**Fig. 4:**
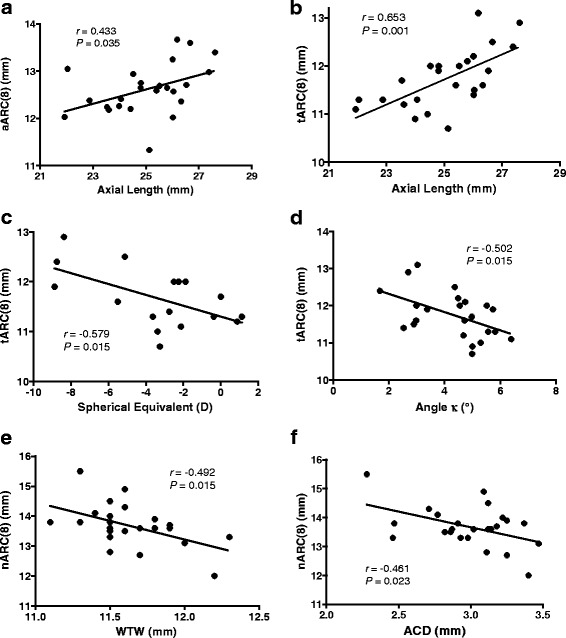
Statistically significant correlations between the scleral curvature and other ocular biometric parameters. **a** Correlation between axial length and average axial radius of curvature at 8 mm to the axis line (aARC[8]). **b** Correlation between axial length and temporal ARC[8] (tARC[8]). **c** Correlation between spherical equivalent and tARC[8]. **d** Correlation between angle kappa and tARC[8]. **e** Correlation between WTW and nasal ARC[8] (nARC[8]). **f** Correlation between ACD and nARC[8]. *r* = Pearson correlation coefficient

The mean value and range of the ages and ocular biometric parameters of the 24 involved eyes are listed in Table 1. Among the parameters, only axial length showed a significant correlation with aARC[8] (Pearson correlation coefficient *r* = 0.433, *P* = 0.035 by Pearson correlation analysis, Fig. 4a). When the nARC[8] and tARC[8] were analyzed separately, they showed completely different correlation patterns (Table 2). The tARC[8] showed statistically significant correlations with axial length (Pearson’s *r* = 0.653, *P* = 0.001, Fig. 4b), spherical equivalent (Pearson’s *r* = −0.579, *P* = 0.015, Fig. 4c), and angle kappa (Pearson’s *r* = −0.502, *P* = 0.015, Fig. 4d). The nARC[8] showed statistically significant correlations with WTW (Pearson’s *r* = −0.492, *P* = 0.015, Fig. 4e) and ACD (Pearson’s *r* = −0.461, *P* = 0.023, Fig. 4f).

**Table 2:** Correlation between scleral curvature and other ocular biometric parameters including age

Parameters	tARC[8]		nARC[8]		aARC[8]	
	Pearson’s *r*	*P*-value (two-tailed)	Pearson’s *r*	*P*-value (two-tailed)	Pearson’s *r*	*P*-value (two-tailed)
Age	−0.197	0.356	−0.141	0.511	−0.199	0.351
Sim Kmax	−0.216	0.406	−0.055	0.833	−0.163	0.532
Sim Kmin	−0.380	0.133	−0.154	0.554	−0.327	0.201
SE	−0.579	0.015*	−0.52	0.843	−0.396	0.116
WTW	−0.166	0.438	−0.492	0.015*	−0.360	0.084
Thinnest corneal thickness	−0.292	0.256	−0.126	0.631	−0.258	0.317
ACD	−0.116	0.590	−0.461	0.023*	−0.310	0.140
Angle Kappa	−0.502	0.015*	0.058	0.792	−0.301	0.163
Axial length	0.653	0.001**	−0.018	0.934	0.433	0.035*

*tARC[8]* axial radius of curvature at 8 mm temporal to the axis line, *nARC[8]* axial radius of curvature at 8 mm nasal to the axis line, *aARC[8]* average values of tARC[8] and nARC[8], *Sim Kmax* maximum simulated keratometric value, *Sim Kmin* minimum simulated keratometric value, *SE* spherical equivalent, *WTW* white-to-white corneal diameter, *ACD* anterior chamber depth from endothelium

*P <0.05, **P <0.01, Pearson’s correlation analysis

### Models for estimating scleral curvature

The results of the univariate and multivariate linear regression analysis for tARC, nARC, and aARC are listed in Table 3. As a result, the following formulae of the best-fit model were developed for *d* = 7.0, 7.5, 8.0, 8.5, and 9.0 mm.

$$ \mathrm{tARC}(d)=-0.846+0.225\times \left(\mathrm{axial}\ \mathrm{length}\right)+0.860\times d $$

(Adjusted coefficient of determination (*R* ^2^_a_) = 0.718, Standard error of the estimate (Std. Error) = 0.454, *P* <0.001)

$$ \mathrm{nARC}(d)=15.039-0.947\times \left(\mathrm{W}\mathrm{T}\mathrm{W}\right)-0.756\times \left(\mathrm{A}\mathrm{C}\mathrm{D}\right)+1.486\times d $$

(*R* ^2^_a_ = 0.796, Std. Error = 0.568, *P* <0.001)

$$ \mathrm{aARC}(d)=8.507+0.124\times \left(\mathrm{axial}\ \mathrm{length}\right)-0.686\times \left(\mathrm{W}\mathrm{T}\mathrm{W}\right)+1.118\times d $$

(*R* ^2^_a_ = 0.767, Std. Error = 0.466, *P* <0.001)

**Table 3:** Univariate and Multivariate Analysis of ARC

Curvature	Input variables	Unit	Univariate analysis			Multivariate analysis		
			Beta coefficient	Std. error	*P*-value	Beta coefficient	Std. error	*P*-value
tARC	*d*	Per mm increase	0.846	0.079	<0.001	0.860	0.072	<0.001
	Axial length	Per mm increase	0.258	0.046	<0.001	0.225	0.029	<0.001
	SE	Per D increase	−0.109	0.028	<0.001			
	Angle kappa	Per ° increase	−0.250	0.061	<0.001			
	Sim Kmin	Per D increase	−0.168	0.066	0.013			
nARC	*d*	Per mm increase	1.486	0.089	<0.001	1.486	0.073	<0.001
	WTW	Per mm increase	−1.186	0.405	0.004	−0.947	0.197	<0.001
	ACD	Per mm increase	−1.015	0.378	0.008	−0.756	0.183	<0.001
aARC	*d*	Per mm increase	1.123	0.069	<0.001	1.118	0.071	<0.001
	Axial length	Per mm increase	0.153	0.057	0.008	0.124	0.030	<0.001
	WTW	Per mm increase	−0.666	0.313	0.036	−0.686	0.167	<0.001
	SE	Per D increase	−0.069	0.034	0.044			

*ARC* axial radius of curvature, *Std. Error* standard error of the estimate, *d* distance from the axis line, *SE* spherical equivalent, *Sim Kmin* minimum simulated keratometric value, *WTW* white-to-white corneal diameter, *ACD* anterior chamber depth from endothelium

### Subgroup analysis for wider scleral areas (*d* = 7.0, 7.5, 8.0, 8.5, and 9.0 mm)

**Fig. 5:**
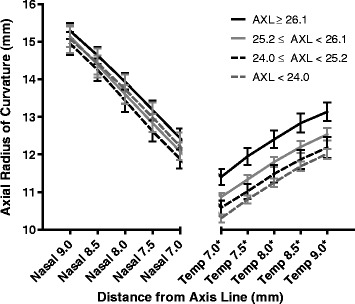
The effect of axial length on axial radius of curvature (ARC) in different points of the sclera. When the 24 eyes were grouped according to axial length (<24.0, 24.0 − 25.2, 25.2 − 26.1, and ≥26.1 mm; *n* = 6 for each group), only the mean ARC in each of the five temporal scleral points showed significant differences (temporal 7.0 mm: *P* = 0.001; temporal 7.5 mm: *P* = 0.001; temporal 8.0 mm: *P* = 0.002; temporal 8.5 mm: *P* = 0.004; temporal 9.0 mm: *P* =0.008). No significant differences were found among the five nasal scleral points. *AXL* axial length; *Temp* temporal. *P <0.05, One-way analysis of variance test

When the 24 eyes were grouped according to axial length (<24.0, 24.0 − 25.2, 25.2 − 26.1, and ≥26.1 mm; *n* = 6 for each group), only the mean scleral curvature in all five temporal scleral points showed significant differences (temporal 7.0 mm: *P* = 0.001; temporal 7.5 mm: *P* = 0.001; temporal 8.0 mm: *P* = 0.002; temporal 8.5 mm: *P* = 0.004; temporal 9.0 mm: *P* =0.008, one-way ANOVA; Fig. 5). No significant differences were found in any of the five nasal scleral points that were analyzed.

The subgroup analysis for the WTW (<11.6 and ≥11.6 mm; *n* = 12 for each group) and ACD (<2.85, 2.85 − 3.05, 3.05 − 3.20, and ≥3.20 mm; *n* = 6 for each group) did not show any statistically significant differences in either the nasal or the temporal sclera.

### Changes in coefficient of determination (*R*^2^) according to distance from the axis line

The *R* ^2^ of the correlation between the axial length and aARC gradually increased, and that between the WTW and aARC gradually decreased as the distance from the axis line increased, which means that in the peripheral portions of the sclera, the scleral curvature correlates more with the axial length and less with the WTW (Table 4).

**Table 4:** Coefficient of determination (*R* ^2^) along the distance from the axis line

Distance from the axis line	Axial length vs aARC		WTW vs aARC	
	*R* ^2^	*P*-value	*R* ^2^	*P*-value
7.0 mm	0.165	0.049*	0.175	0.042*
7.5 mm	0.176	0.041*	0.156	0.056
8.0 mm	0.187	0.035*	0.130	0.084
8.5 mm	0.203	0.027*	0.102	0.129
9.0 mm	0.230	0.017*	0.069	0.217

*aARC* average axial radius of curvature

*P < 0.05, Pearson’s correlation analysis

## Discussion

AS-OCT has been used to measure the anterior segment, anterior chamber, and angle biometry including corneal pachymetry, corneal diameter, corneal sagittal height, corneoscleral junction angle, scleral curvature, angle-to-angle width, iris diameter, ACD, angle opening distance, angle recess area, trabeculo-iris space area, and anterior chamber angle in degrees [5, 14, 15]. For scleral curvature, the tangential radius of curvature can be measured only by using built-in caliper and protractor tools [5, 6]. The authors developed a method for calculating the axial radius of curvature, which is more clinically useful than a tangential curvature [9]. Although the previous manual method showed excellent reliability and accuracy, the procedure is time-consuming and requires extensive training for usage, expensive imaging devices (AS-OCT), and expensive image processing software (Image-Pro Plus). Two contrasting approaches were used in this study to solve these problems. The first approach was establishing an easier and automated method for curvature analysis using more-sophisticated tools. These efforts resulted in the methods used in this study, which were semi-automated image composition using the ImageJ software with the MosaicJ plug-in, and automated mathematical analysis of the ARC using MATLAB software. However, the outermost surface beyond the limbus is actually the conjunctiva, not the sclera. We held an underlying assumption that the scleral surface is parallel to the conjunctival surface. To satisfy this assumption, eyes with previous and current conjunctival diseases including degenerative changes, a history of previous conjunctival surgery, and current contact lens wearers were excluded from this study. Moreover, the AS-OCT images were manually checked for significant discrepancies. Age did not show a significant correlation with ARC in this study, possibly because of the exclusion criteria; age was reported to have a significant effect on tangential scleral curvature, possibly because of the accumulation of fatty deposits such as pingueculae [5]. Regardless, minor discrepancies between the conjunctival and scleral curvature can exist and could possibly limit this method. Another limitation is that only horizontal ARC could be determined with this method. We attempted this process with vertical images but failed because of difficulties in acquiring wide images due to the tightness of the eyelid in our Asian volunteers. However, on the basis of the results of previous studies on tangential curvature, we can assume that the superior and inferior curvatures lie within the temporal and nasal curvatures [5, 6].

The second approach entailed developing formulae to estimate the scleral curvature using more easily available ocular biometric parameters. As a result, the formulae to estimate the scleral curvature were developed as a function of axial length, ACD, WTW, and distance from the axis line with good determinant power (72 − 80 %). The formulae were developed for the wider area of the sclera (7.0, 7.5, 8.0, 8.5, and 9.0 mm from the axis line) to be compatible with various diameter scleral lenses and to assist the design of the multi-curve haptic of the scleral lenses. As a limitation, the formulae themselves cannot be directly applied to the eyes of non-Asian groups because ocular biometric parameters vary widely according to ethnic groups [16]. However, the relationship between these parameters may not actually change much according to the ethnic groups. For example, a scleral lens with a flatter haptic (scleral part) design can be tried as an initial trial lens for an eye with longer axial length or smaller WTW. With this ocular-parameter-based approach for the selection of an initial trial lens, patients can benefit by the decreased discomfort and anxiety caused by a poorly fitted initial trial lens and the clinician can benefit by decreased chair time for fitting. However, these formulae could be validated more firmly by comparing the predicted ARC values with the analyzed values in an independent cohort.

**Fig. 6:**
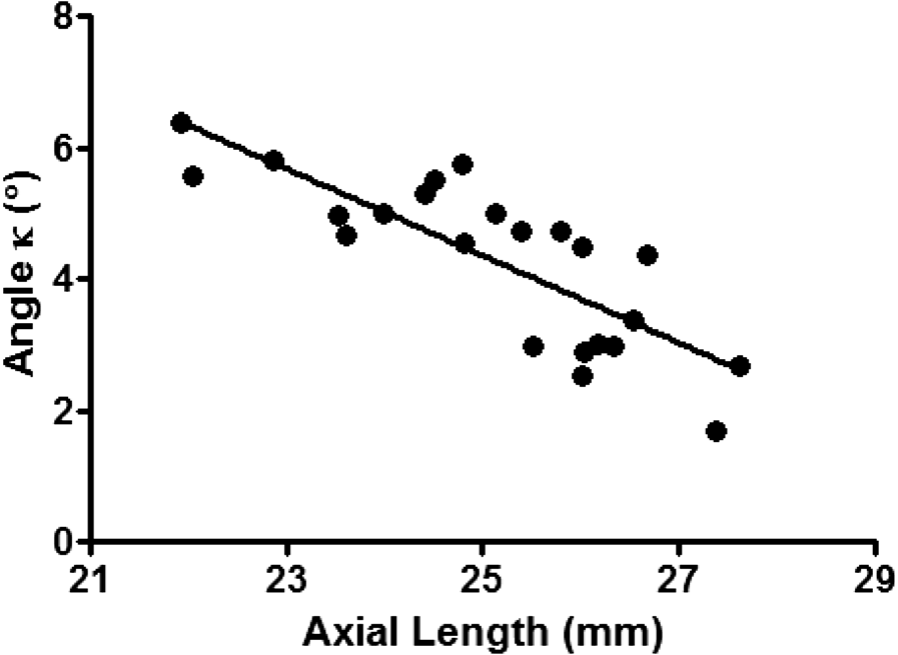
Correlation between axial length and angle kappa. Angle kappa showed a strong correlation with axial length (Pearson’s *r* = −0.813 and *P* <0.001), stronger than the spherical equivalent correlation (Pearson’s *r* = 0.685 and *P* = 0.003)

In this study, axial length, ACD, and WTW were found to be useful parameters for estimating scleral curvature. In the univariate linear regression analysis, the temporal scleral curvature showed significant correlations with axial length, spherical equivalent, angle kappa, and minimum Sim K. However, only axial length remained as a significant parameter for the model-fitting in the multivariate regression analysis owing to the collinearity caused by the strong correlation between axial length and spherical equivalent (Pearson’s *r* = −0.828, *P* <0.001), angle kappa (Pearson’s *r* = −0.813, *P* <0.001, Fig. 6), and minimum Sim K (Pearson’s *r* = −0.635, *P =* 0.006). By contrast, the nasal scleral curvature showed significant correlations with WTW and ACD in the univariate and multivariate linear regression analyses (Table 3). To summarize, temporal scleral curvature correlated with axial length, and nasal scleral curvature correlated with ACD and WTW independently.

Increased axial length can affect the scleral curvature by leading to an accompanying increase in the width (coronal dimension) of the eyeball [17]. We expected ACD to correlate well with scleral curvature because ACD is partially determined according to corneal curvature, particularly the peripheral corneal curvature that is just adjacent to the scleral curvature [18]. However, the predictive power itself was relatively low. ACD did not show any significant correlation with maximum Sim K (Pearson’s *r* = 0.255, *P* = 0.323), minimum Sim K (Pearson’s *r* = 0.244, *P* = 0.344), WTW (Pearson’s *r* = 0.294, *P* = 0.163), or axial length (Pearson’s *r* = 0.215, *P* = 0.314), contrary to several previous reports [19–21]. However, similar results were reported in a large population-based cross-sectional study, which alludes to the complexity of ACD determination [15]. The scleral curvature is flatter than the corneal curvature and becomes more flat with the increase in the distance from the axis line (Figs. 2 and 3). Hence, the curvature of the sclera can be affected by the WTW because the distance from the limbus (transition zone) varies according to the WTW. The nasal sclera showed correlation only with the WTW, which may be caused by the larger change in curvature in the nasal limbus compared with the temporal limbus (Fig. 2, bottom left) [5, 6, 9]. However, the correlation disappears rapidly with increasing distance from the axis line (Table 4). Ocular parameters affect scleral curvature in different patterns according to the distance from the axial line as shown in Table 4. A significant correlation was observed between WTW and aARC only at the point where *d* is 7.0 mm (Table 4). This could possibly explain the reason why WTW was added to the formula of aARC(*d*), even though no significant correlation was found between WTW and aARC[8]. The WTW did not show any significant correlation with axial length (Pearson’s *r* = −0.054, *P* = 0.802) and ACD (Pearson’s *r* = 0.294, *P* = 0.163).

Two interesting findings were observed with regard to angle kappa in this study. The first finding was the strong relationship between angle kappa and axial length (Pearson’s *r* = −0.813, *P* <0.001; Fig. 6). Angle kappa is defined as the angle between the pupillary axis (the line perpendicular to the cornea that passes through the center of the entrance pupil) and the visual axis (line connecting the fixation point with the fovea), although it can be also referred to as angle lambda [22, 23]. Angle kappa has clinical significance in measuring the angle of strabismus and the centration issues during refractive surgeries [23–25]. In addition, angle kappa has recently drawn attention for its potential influence on the effect of complex optical devices such as multifocal intraocular lenses and on postoperative visual satisfaction [26, 27]. Several clinical reports have shown that hyperopic or emmetropic eyes have a tendency to have a larger angle kappa than myopic eyes owing to the clinical experiences of the refractive surgeries [23–25, 28]. However, there are conflicting results regarding the value of angle kappa between emmetropic and hyperopic eyes [23, 24]. In our study, the angle kappa showed stronger correlation with axial length (Pearson’s *r* = −0.813, *P* <0.001) than with spherical equivalent (Pearson’s *r* = 0.685, *P* = 0.003). This study suggests that axial length is a more appropriate predictor of angle kappa than spherical equivalent, which has been studied so far. However, a larger-scale study is necessary to confirm this suggestion.

The second interesting finding was that the different correlation patterns of nasal and temporal scleral curvatures with axial length can be mediated by angle kappa. The nasal scleral curvature is flatter (with a larger ARC) than the temporal sclera, which can be partially explained by the sharp corneoscleral junction angle in the nasal meridian [5, 6]. However, this could not fully explain why the axial length shows completely different correlation patterns with the nasal and temporal sclera.

**Fig. 7:**
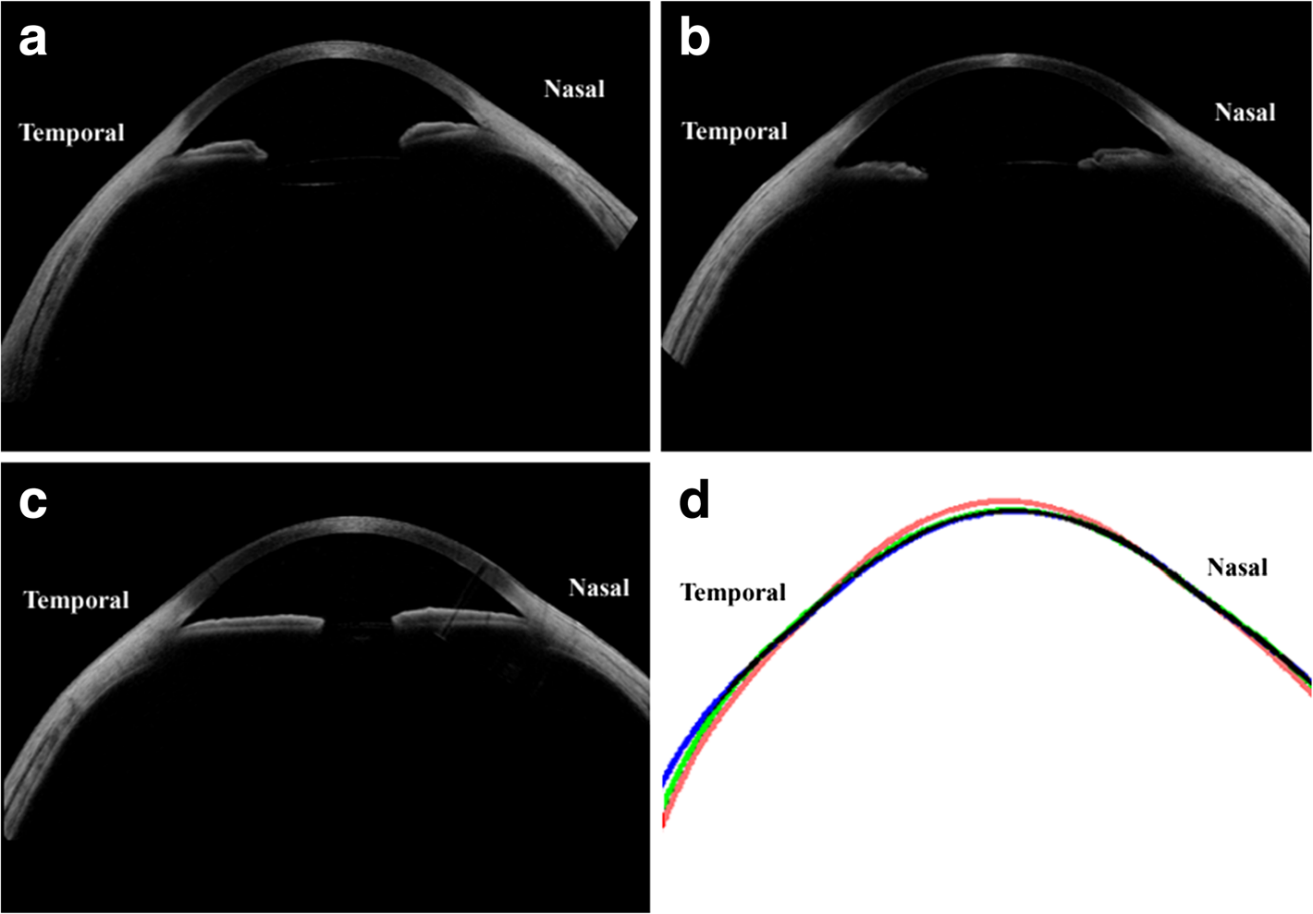
Ocular surface alignment (angle kappa) and scleral curvature changes along with axial length. **a** The composite anterior segment tomographic image of an eye with shorter axial length (21.93 mm) and larger angle kappa (6.39°); the extracted ocular surface outline from this image is the *red line* in **d. b** The composite image of an eye with medium axial length (25.40 mm) and medium angle kappa (4.72°); the extracted ocular surface outline is the *green line* in **d. c** The composite image of an eye with longer axial length (27.38 mm) and smaller angle kappa (1.68°); the extracted ocular surface outline is the *blue line* in **d. d** The overlap of the lines extracted from the outermost layer of the ocular surface using the MATLAB software. The *red line* represents the eyes with shorter axial length (Fig. 7a), the *green line* represents medium axial length (Fig. 7b), and the blue line represents longer axial length (Fig. 7c). The nasal scleral curvature flattens considerably less than the temporal side owing to the flattening of the scleral curvature with longer axial length, counteracted by the steepening effect of the accompanying decreased angle kappa

A likely explanation may be provided by considering the relationship between angle kappa and axial length. The composite images of the anterior surface in this study show that angle kappa is the angle between the visual axis and total anterior segment, including the cornea, iris, lens, and sclera, as shown in Fig. 7. The angle kappa is actually a misalignment between the functional axis of the eye, which is represented by the “visual axis,” and the anatomical axis of the anterior eye, which is represented by the “pupillary axis” [22]. If the anterior surface shows no deviation, both the nasal and temporal sclera should be flattened (increasing both nARC and tARC) together with the increased axial length. However, the positive angle kappa induces flatter curvature in the nasal sclera (higher nARC) and steeper curvature in the temporal sclera (lower tARC). In the nasal sclera, the direct flattening effect (thereby increasing nARC) induced by the longer axial length is compensated by the indirect steepening effect (thereby decreasing nARC) by the accompanying decreased angle kappa. Meanwhile, in the temporal sclera, the direct flattening effect by the longer axial length is enhanced by the indirect flattening effect by the decreased angle kappa. This synergy resulted in a stronger correlation between the temporal scleral curvature and the axial length. The axial length showed relatively constant significant correlations with curvature in the wider-range temporal sclera (*d* = 7.0, 7.5, 8.0, 8.5, and 9.0 mm), but not in the wider-range nasal sclera (Fig. 5).

Another possible hypothesis for this difference is the asymmetric growth of sclera during the myopic eye growth. If the temporal sclera is assumed to expand more than the other quadrants, the close correlation of axial length with the temporal scleral curvature and angle kappa can be explained simultaneously. This hypothesis can be supported by the tangential curvature differences between the temporal sclera and the other quadrants [5, 6]. However, the scope of this study is limited to the anterior sclera, and a well-designed follow-up study is required to prove this hypothesis, including the analyses of the shape of the posterior sclera and longitudinal observation.

## Conclusions

The authors developed an automated method of calculating the axial curvature of the anterior sclera, and formulae to estimate the scleral curvature by incorporating axial length, ACD, WTW, and the distance from the axis line, with good determinant power (72 − 80 %). In addition, angle kappa that represents ocular surface alignment was rediscovered in this study for its strong correlation with axial length, and for its notable role in the discrepancy between the nasal and the temporal sclera. We hope these results can deepen our knowledge on the anatomical and functional misalignments of the anterior eye and provide practical information for designing and fitting of scleral lens haptics.

## Additional file

Additional file 1: Figure S1. The process of making a composite image covers a wide area of the anterior ocular surface from three horizontal tomographic images. After rotating the nasal and temporal horizontal images using the image rotate function of ImageJ, the composite image is created semi-automatically using the MosaicJ plugin of ImageJ. (TIF 1672 kb)

## Abbreviations

ACD: Anterior chamber depth (from endothelium)
ARC: Axial radius of curvature
tARC: Temporal ARC
tARC[8]: ARC at 8 mm temporal to the axis line
nARC: Nasal ARC
nARC[8]: ARC at 8 mm nasal to the axis line
aARC: Average ARC
aARC[8]: Average values of tARC[8] and nARC[8]
AS-OCT: Anterior segment optical coherence tomography
*d*: Distance from the axis line
SE: Spherical equivalent
Sim Kmax: Maximum simulated keratometric value
Sim Kmin: Minimum simulated keratometric value
Std. Error: Standard error of the estimate
WTW: White-to-white corneal diameter
